# Exploring the patterns of availability and provision of sexual and reproductive health services to young people in primary healthcare centers in Ebonyi state, Nigeria

**DOI:** 10.1186/s12913-025-13208-4

**Published:** 2025-08-11

**Authors:** Joy Nkeiruka Ozughalu, Ozioma Patricia Nwankpa, Chinyere Ojiugo Mbachu, Obinna Onwujekwe

**Affiliations:** 1 Health Policy Research Group, Department of Pharmacology and Therapeutics, College of Medicine, University of Nigeria, Enugu Campus, Enugu, Nigeria; 2 Department of Community Medicine, College of Medicine, University of Nigeria, Enugu Campus, Enugu, Nigeria; 3 Institute of Public Health, College of Medicine, University of Nigeria, Enugu Campus, Enugu, Nigeria; 4 Department of Health Administration and Management, Faculty of Health Sciences and Technology, University of Nigeria, Enugu Campus, Enugu, Nigeria

**Keywords:** Sexual and reproductive health (SRH), Health service, Availability, Provision, Adolescent, Young people

## Abstract

**Background:**

Adolescents and young people represent a significant portion of the population in Nigeria. Thus, addressing their unique sexual and reproductive health (SRH) needs is imperative for overall national health outcomes, especially given the limited availability of youth friendly services. This study aimed to identify the factors that influence availability and provision of SRH services to young persons in Ebonyi State, Southeast Nigeria.

**Methods:**

A cross-sectional quantitative study was used to explore the patterns of health service availability and provision among healthcare providers in primary healthcare centers. Descriptive analysis was used to examine respondents’ socio-demographic characteristics and their provision of differential treatment based on gender and age. Rao-Scott chi-squared test assessed the relationship between the two outcome variables-health service availability and provision. Multiple logistic regression, adjusted for clustering at the facility level, was used to examine associations between dependent variables and independent variables, including age, gender, location, years of formal education, and training in providing youth-friendly sexual and reproductive health services.

**Results:**

Rural areas reported higher service availability than urban areas, likely due to targeted national health programs. However, service provision remained inconsistent, especially for post-abortion care and support for survivors. Higher provider education was positively linked to both availability and provision. Youth-friendly training improved reported availability but did not enhance actual service delivery.

**Conclusion:**

While targeted interventions have improved service availability in rural areas, significant gaps remain in actual service provision, particularly for sensitive SRH services. Enhancing provider education and addressing systemic barriers to youth-friendly care are essential to translating availability into meaningful access and quality service delivery.

**Supplementary Information:**

The online version contains supplementary material available at 10.1186/s12913-025-13208-4.

## Introduction

In Nigeria, where young people aged 10–24 represent 32% of the population [1], addressing their unique sexual and reproductive health (SRH) needs is imperative for overall national health outcomes [2]. SRH encompasses a broad spectrum of services and information, including family planning, sexually transmitted infection (STI) prevention and treatment, and maternal health care, that are crucial for individuals to achieve optimal health and well-being [3]. For young people, particularly in developing regions, access to SRH services is essential for promoting healthy behaviors, preventing STIs, and reducing unintended pregnancies [4].

Despite national policies aimed at improving SRH outcomes, the availability and provision of these services remain inadequate in many parts of the country. With only about 20% of Primary Health Centers (PHCs) in Nigeria fully functional [5], these centers often struggle to maintain a consistent supply of essential SRH commodities, such as contraceptives, HIV test kits, and STI medications, making it difficult to deliver comprehensive care and limiting access for young people [6]. This inconsistency also hampers the ability to deliver timely SRH services, undermining efforts to reduce rates of unintended pregnancies, prevent the spread of infections, and build trust in the healthcare system.

Moreover, the availability of SRH services does not automatically translate to access. Factors such as distance to health facilities, cost of services, and confidentiality concerns significantly influence whether young people utilize available services. Provider hesitancy to offer SRH services to adolescents in Nigeria is influenced by factors such as inadequate training, age-related biases, and prevailing cultural and religious norms [7, 8]. These issues often result in judgmental attitudes or outright denial of care, particularly toward unmarried youth, further limiting their access to essential SRH services and creating barriers to open discussion [9–11].

Systemic issues, including understaffing and lack of youth-specific training, compound these challenges. A study assessing healthcare workers’ delivery of adolescent-responsive sexual and reproductive healthcare services in Plateau state revealed that most healthcare workers had not received prior training on adolescent sexual and reproductive health [12]. These barriers exacerbate poor SRH outcomes, with Nigeria recording an adolescent birth rate of 106 per 1,000 females aged 15–19, among the highest globally (United Nations Population Fund [13].

These studies highlight the urgent need for policy implementation and capacity-building interventions that address both attitudinal and structural barriers to the provision of SRH services for young people in Nigeria. However, a clearer understanding of the baseline realities is essential before designing effective and context-appropriate interventions. This study, therefore, seeks to examine the current landscape of SRH service availability and provision for young people in primary healthcare centers, assessing whether these services are genuinely accessible and delivered by healthcare providers. Additionally, it explores the key factors that facilitate or hinder the availability and delivery of these services in Ebonyi State, Nigeria.

## Methods

### Study design and area

The cross-sectional study was undertaken in Ebonyi state, southeast Nigeria. The state has an estimated population of 4,339,136, with a yearly growth rate of 2.8%. Over 355,000 of its residents are young people between the ages of 15–24 [14, 15]. This demographic concentration underscores the importance of understanding service availability and provision, as intervening early can shape the lifelong health behaviors of young people, reducing the burden of STIs, HIV, and unintended pregnancies [16].

The study was conducted across six local government areas (LGAs) in Ebonyi State, Nigeria. These were the six LGAs with the poorest SRH outcomes for young people. These were Abakaliki and Izzi in Ebonyi North senatorial zone, Ikwo and Ezza South in Ebonyi Central senatorial zone, and Afikpo South and Ohaozara in Ebonyi South senatorial zone. These six LGAs contain 84 primary health centers (PHCs) that are designated as youth-friendly health centers. These facilities are prioritized by the state government and partners for scaling up of SRH services.

### Study population and sampling

The target population for this study consisted of healthcare workers actively providing SRH services to young people in selected PHCs across six the LGAs in Ebonyi State. A total of 84 youth-friendly PHCs were purposively selected for the study. Using Cochran’s formula for infinite populations, we first calculated the initial sample size (n₀) as follows:

$$n_0=\frac{\left(Z^2\times p\times\left(1-p\right)\right)}{\alpha^2}$$

where, Z is the Z-score corresponding to the desired confidence level (1.96 for 95% confidence), p is the estimated proportion of healthcare providers that are providing SRH services in the population (0.5), and α is the desired margin of error (0.05). To adjust for a finite population of size N, the Cochran’s modified formula:

$$n=\frac{n_0}{\left[1+\left({\displaystyle\frac{\left(n_0-1\right)}N}\right)\right],}$$

where N = estimated population of PHC workers in the selected LGAs (700) was used.

Thus, the minimum required sample size was 249 healthcare workers. Based on prior field experience and findings from a pilot study involving healthcare providers, we anticipated a high level of cooperation during data collection. Given this expectation, along with the brief duration of interviews, no additional adjustment was made for potential attrition.

Three eligible health workers from 84 PHCs who met the criteria of providing SRH services to young people were selected for the survey. In centers where there were more than three eligible health workers, respondents were recruited on a consecutive basis resulting in a total of 263 health workers from this study. However, due to missing responses to key survey questions on SRH service provision the usable sample size was reduced to 255 health workers.

### Study tool and data collection

Data collection was carried out using an interviewer-administered, pretested questionnaire, which was originally adapted from UN Women’s 2013 annual publication on gender and evaluation and the Compendium of Gender Scales [17, 18]. However, this present study focused on a specific aspect of the questionnaire that was not part of the model adapted from these sources. The final questionnaire that was used for this study has been uploaded as a supplementary file.

Information was collected from healthcare providers, including their socio-demographic characteristics and their responses regarding the availability and provision of sexual and reproductive health services for adolescents and young people. In order to ascertain the health services that were available and provided by health workers in the selected facilities, the questions “Which of the following health services are available in this facility?” and “Which of the following health services have you provided in this facility?” were asked. Respondents could select all applicable options from a provided list (seen in Table 1). Each service option was coded as ‘1’ if the service was available or provided, and ‘0’ if it was not available or provided.

**Table 1:** Items analyzed for services available and services provided

1. Information and counseling on reproductive health service	9. Care after child birth
2. HIV testing and counseling	10. Post abortion care
3. HIV care and support	11. Information and counseling on contraception emergency
4. Diagnosis of STIs/RTIs	12. Information and counseling on condoms and condom use
5. Treatment and counseling for STIs/RTIs	13. Supply of contraceptives condoms
6. Pregnancy testing	14. Care and support for physical abuse
7. Care during pregnancy	15. Care and support for sexual abuse/rape
8. Care during childbirth	

### Data analysis

Descriptive analysis was conducted to summarize the socio-demographic characteristics of the respondents. To assess the relationship between the two binary outcome variables - health service availability and health service provision -, a Rao Scott’s chi-square test was performed, and the strength of their association was measured using Cramer’s V. Given the nature of the data, with respondents nested within health facilities, clustering at the facility level was accounted for in all inferential analyses. Specifically, logistic regression models were fitted to examine the association between each dependent variable (availability or provision of services) and independent variables such as age, gender, location, years of formal education, and whether the respondent had received formal training in the provision of youth-friendly sexual and reproductive health services. Robust standard errors were calculated to adjust for clustering at the facility level, ensuring valid statistical inference.

**Fig. 1:**
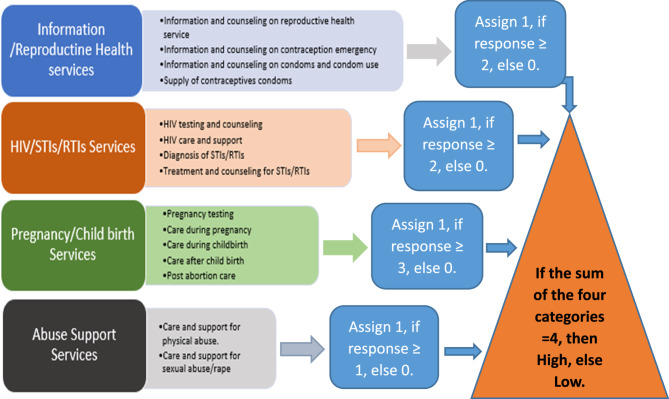
Transformation framework of service availability/provision items

To streamline the analysis of provider-reported data on sexual and reproductive health (SRH) services, we developed a simplified coding framework that grouped 15 individual service items into four conceptually aligned domains: Information/Reproductive Health Services, HIV/STIs/RTIs Services, Pregnancy/Childbirth Services, and Abuse Support Services (see Table 1; Fig. 1).

Each domain consisted of related services: Information/Reproductive Health Services included information and counseling on reproductive health, emergency contraception, condom use, and the supply of contraceptive condoms. These represent key informational and preventive services supporting informed sexual health choices.

HIV/STIs/RTIs Services covered HIV testing and counseling, HIV care and support, diagnosis of STIs/RTIs, and treatment and counseling for these conditions. These services function collectively to address infection prevention, diagnosis, and treatment.

Pregnancy/Childbirth Services encompassed pregnancy testing, care during pregnancy, childbirth, postnatal care, and post-abortion care—reflecting a continuum of maternal and reproductive care.

Abuse Support Services comprised care and support for both physical abuse and sexual abuse or rape, recognizing the importance of addressing gender-based violence as part of comprehensive SRH services.

To classify adequacy within each domain, binary thresholds were applied based on the number of services present. In the Information/Reproductive Health Services and HIV/STIs/RTIs Services domains, the presence of two or more services was considered “High” (coded as 1); otherwise, it was “Low” (coded as 0).

For Pregnancy/Childbirth Services, the threshold was three or more services to be classified as “High.”. For the Abuse Support Services domain, the presence of both services was required to classify it as “High”; otherwise, it was considered “Low.”

Each domain was coded as either “High” or “Low” in terms of service availability and provision. The binary values across all four domains were summed to generate an overall adequacy score. A total score of four indicated “Adequate or High” overall service availability or provision, while scores below four were classified as “Inadequate or Low.”

This method provided a meaningful and interpretable summary measure of SRH service adequacy, while still preserving the underlying structure and detail of the individual service items. While this framework is a novel contribution, it is conceptually informed by established practices in composite index construction in global health research. Millogo et al. [19] used grouped service indicators and binary scoring logic to evaluate facility readiness, supporting the use of similar methods in provider-reported service analysis.

### Ethical approval

This study was conducted in accordance with the principles of the 1964 Declaration of Helsinki and ethical approval was secured from the Health Research Ethics Committee of the University of Nigeria Teaching Hospital Enugu, with reference number: UNTH/NREC/2024/01/335. A written informed consent form was obtained from all respondents prior to their participation in the study. Participation was entirely voluntary, with respondents having the right to withdraw at any stage without any consequences. Strict confidentiality measures were implemented to protect participants'identities and ensure that all collected data remained anonymous and used solely for research purposes

## Result

In table 2, univariate analysis revealed that 90.6% of participants were female, with 37.3% residing in rural areas. Only about 5% had educational attainment exceeding seven years. Approximately 73% of respondents were Community Health Workers, while 92.5% of respondents had received training specifically on youth-friendly sexual and reproductive health services. Over 95% of these health workers reported treating boys and girls, as well as youth and adults, without differentiation in care practices.

**Table 2:** Socio-demographic and behavioral characteristics of health service providers

	Frequency *N* = 255	Percent (%)
Gender		
• Male	24	9.4
• Female	231	90.6
Location		
• Urban	95	37.3
• Rural	160	62.7
Age		
• Mean (Standard deviation)	**34.18 (10.40)**	
Years of Formal Education Received for Current Work		
• Group 1 (0–6years)	235	92.2
• Group 2 (7–12years)	20	7.8
• Mean (Standard deviation)	**3.224 (2.11)**	
Current Role As A Provider		
• Community health workers	186	72.9
• Officer in Charge	46	18.0
• Frontline health worker in YF-centers	16	6.3
• Adolescent health Focal Officer	6	2.4
• Youth councilor	1	0.4
Received Formal Education for Provision of Youth-Friendly SRH Services	236	92.5
Total Years of Education		
• Mean (Standard deviation)	**15.05 (2.40)**	
Providing different treatment for boys vs. girls	11	4.3
Providing different treatment for Youth Vs Adults	12	4.7

Table 3 shows that while 84.7% of providers reported availability of HIV care and support, only 52.2% actively provided them. Similarly, while 86.7% of health workers indicated availability of diagnostic services for sexually transmitted infections (STIs) or reproductive tract infections (RTIs), only 44.7% delivered these services. Among the assessed services, post-abortion care was the least frequently provided, despite 96.1% of respondents reporting its availability. Furthermore, care and support services for individuals experiencing physical and sexual abuse were found to be inadequately provided.

**Table 3:** Descriptive analysis of SRH services available and provided for young people by healthcare workers in phcs

	HEALTH SERVICES AVAILABLE		HEALTH SERVICES PROVIDED	
	YES (%)	NO (%)	YES (%)	NO (%)
Information and counselling on reproductive health, sexuality & safe sex	235(92.2)	20 (7.8)	216(84.7)	39(15.3)
HIV testing and counseling	231 (90.6)	24 (9.4)	221(86.7)	34(13.3)
HIV care and support	216 (84.7)	39 (15.3)	133(52.2)	122(47.8)
Diagnosis of STI/RTI	221 (86.7)	34 (13.3)	114(44.7)	141(55.3)
Treatment and counseling for STIs/RTIs	133 (52.2)	122 (47.8)	177(69.4)	78(30.6)
Pregnancy testing	114 (44.7)	141 (55.3)	244(95.7)	11(4.3)
Care during pregnancy	177 (69.4)	78 (30.6)	247(96.9)	8(3.1)
Care during childbirth	244 (95.7)	11 (4.3)	245(96.1)	10(3.9)
Care after childbirth	247 (96.9)	8 (3.1)	243(95.3)	12(4.7)
Post-abortion care	245 (96.1)	10 (3.9)	100(39.2)	155(60.8)
Information & counseling on contraception/emergency contraception	243 (95.3)	12 (4.7)	217(85.1)	38(14.9)
Information and counseling on condoms and condom use	100 (39.2)	155 (60.8)	217(85.1)	38(14.9)
Supply of contraceptives/condoms	217 (85.1)	38 (14.9)	229(89.8)	26(10.2)
Care and support for physical abuse	217 (85.1)	38 (14.9)	172(67.5)	83(32.5)
Care and support for sexual abuse (rape)	229 (89.8)	26 (10.2)	163(63.9)	92(36.1)

The study revealed notable disparities in reported health service availability and provision (Table 4). Among the surveyed health providers, 82.4% (*n* = 210) reported high (adequate) service availability, while 17.6% (*n* = 45) indicated low (inadequate) availability. Regarding service provision, 65.5% (*n* = 167) reported high provision of health services, whereas 34.5% (*n* = 88) reported low provision

Further analysis using cross-tabulation showed a clear pattern: among those reporting high service availability, 71% (*n* = 149) also reported high provision, while 29% (*n* = 61) reported low provision. Conversely, of those reporting low service availability, only 40% (*n* = 18) reported high provision, while 60% (*n* = 27) reported low provision

To test the strength of this relationship while accounting for clustering at the health facility level, a Rao-Scott adjusted chi-square test was conducted. The results indicated a statistically significant association between service availability and provision (F (1, 83) = 11.24, *p* = 0.0013). However, the Cramér’s V value of 0.248 suggests a weak to moderate association.

**Table 4:** Health service availability and provision, and their association

Variable	Category	Frequency (*n*)	Percent (%)
**A. Overall Distribution**			
Health Service Availability	High (Adequate)	210	82.4
	Low (Inadequate)	45	17.6
Health Service Provision	High (Adequate)	167	65.5
	Low (Inadequate)	88	34.5
**B. Cross-tabulation of Availability and Provision**			
	Health Service Provision (HSP)		
Health Service Availability (HSA)	**High (n, %)**	**Low (n, %)**	**Total (n)**
**High (Adequate)**	149 (71.0%)	61 (29.0%)	210
**Low (Inadequate)**	18 (40.0%)	27 (60.0%)	45
Total	167	88	255

Rao-Scott adjusted chi-square test: F(1, 83) = 11.24, *p* = 0.0013

Cramér’s V: 0.248

In Table 5, logistic regression models were fitted to examine factors associated with health service availability and health service provision. Both models adjusted for clustering at the health facility level using cluster-robust standard errors. Urban location was significantly associated with lower odds of high service availability (OR = 0.36, *p* = 0.020), but had no significant association with service provision. Compared to community health workers, other cadre of health providers were significantly less likely to report high service provision (OR = 0.51, *p* = 0.045), and marginally less likely to report service availability (OR = 0.42, *p* = 0.067).

**Table 5:** Logistic regression results for HSAF and HSPF (Cluster-Robust SEs by Facility)

Predictor	OR (HSAF)	95% CI (HSAF)	*p*-value	OR (HSPF)	95% CI (HSPF)	*p*-value
Intercept	2.59	0.05–124.30	0.629	0.07	0.01–0.70	0.024*
Age	1.04	0.99–1.09	0.151	1.02	0.98–1.06	0.309
Urban vs. Rural	0.36	0.15–0.85	0.020*	1.28	0.61–2.67	0.509
Male vs. Female	2.44	0.58–10.26	0.224	0.69	0.25–1.93	0.478
Other role vs. CHW	0.42	0.16–1.06	0.067	0.51	0.26–0.99	0.045*
Trained for Work (Yes)	1.32	0.48–3.66	0.588	1.40	0.48–4.09	0.535
Years Formal Education	1.46	0.96–2.21	0.075	1.21	0.98–1.48	0.070
Total Years of Education	0.89	0.66–1.21	0.466	1.15	0.98–1.34	0.083
Youth-Friendly Training (Yes)	2.32	1.09–4.95	0.029*	0.80	0.39–1.63	0.533

Note: Asterisks (*) indicates statistical significance levels (p < 0.05)

Training in youth-friendly services significantly increased the odds of reporting high service availability (OR = 2.32, *p* = 0.029), but had no effect on service provision. Formal education showed marginal positive associations with both outcomes (HSAF and HSPF), though not statistically significant at the 0.05 level. Age, sex, and general work training were not significantly associated with either outcome.

## Discussion

The analysis reveals important insights into the determinants of health service availability and provision in primary healthcare facilities. Contrary to expectations, the analysis indicates that urban location is associated with lower odds of reporting high service availability compared to rural areas. This counters traditional assumptions of urban advantage, but aligns with evidence suggesting that targeted national initiatives such as Nigeria’s Primary Health Care Under One Roof (PHCUOR) and Maternal, Newborn, and Child Health (MNCH) programs effectively bolster rural service delivery [20, 21], through focused investments, community-based strategies, and donor support. In contrast, urban PHCs are often overlooked due to assumptions of adequacy, yet they face challenges like overcrowding, fragmented governance, and limited external funding. As a result, rural facilities may report higher service availability not because urban areas lack need, but because rural areas have received more structured and sustained support. Programs leveraging community-specific deployment strategies have shown measurable success in rural Nigeria by increasing health worker competency and service uptake [22]. Callaghan et al. [23] similarly observed how external funding and targeted programs can temporarily invert the typical urban-rural disparity

Education remains a critical enabling factor. In both models, years of formal education showed a marginally significant positive association with service availability and service provision. Although not statistically significant at the conventional 0.05 level, these findings reinforce the idea that more educated providers may be better equipped to understand, adopt, and implement complex health interventions. Previous work by Nyblade et al. [24] and Jonas et al. [25] supports this, highlighting education as a mechanism for improving provider competence and patient outcomes, particularly in the delivery of SRH services. In addition, Babalola, Fatusi, and Anyanti [26] found that educated providers are better equipped to handle youth-friendly SRH services and apply critical thinking to navigate cultural or social challenges

Another key determinant of SRH service provision is the cadre of Community Health Workers (CHWs). The analysis shows that healthcare providers who are not classified as CHWs were significantly less likely to report high levels of SRH service provision, underscoring the critical role CHWs play in frontline healthcare delivery. Unlike other provider types, CHWs are often recruited from within the communities they serve, which fosters trust, cultural alignment, and a deeper understanding of local health needs and social dynamics. This embeddedness enables them to deliver patient-centered care more effectively, particularly in sensitive areas like contraceptive counseling and youth engagement [27]. Their ability to navigate cultural norms, communicate in local languages, and maintain close relationships with community members makes them uniquely positioned to overcome barriers that often hinder SRH service uptake [28, 29]

Having undergone youth-friendly training was significantly associated with increased health service availability, but not with improved service provision. This discrepancy suggests that while training may lead to better equipping facilities on paper, it does not necessarily translate to actual delivery of services. This gap may stem from; poor follow-through after training, cultural or religious resistance to youth SRH, lack of institutional support to implement youth-centered practices. This reinforces concerns raised in previous research [30, 31] that training alone- if not values-based or context-sensitive - may fail to change provider behaviors. To overcome these barriers, a holistic approach is required, including continuous mentorship, supportive supervision, and community engagement to increase the provision of SRH services. Only through such comprehensive strategies can training efforts translate into meaningful and sustained improvements in service provision

Neither age nor gender of the provider showed significant associations with either outcome in the adjusted models. Age and experience are often assumed to correlate with better service delivery [9] - as some individuals often preferred older health workers for their perceived wisdom and experience, which can influence patient confidence in SRH services. However, the current data suggest that structural and contextual factors like role, education, and training quality may be more decisive. Envuladu et al. [12] highlighted that younger providers were more adaptable to youth-friendly SRH services, a critical aspect for engaging adolescents, suggesting that age and experience alone may not guarantee better service provision. Similarly, general training (i.e., being trained for the job) was not significantly linked to availability or provision

The significant Rao-Scott adjusted chi-square result strengthens earlier studies that reported that service availability is meaningfully associated with service provision. Improved availability, driven by better resource allocation and staff training, is often closely linked to enhanced service provision. For example, Bintabara, Ernest, and Mpondo [32] demonstrated in Tanzania that improving service availability and readiness, such as ensuring adequate infrastructure and trained personnel, significantly increased SRH service utilization, particularly in underserved communities. Similarly, Tumwine et al. [33] found that investments in SRH infrastructure in Uganda led to increased antenatal care and family planning consultations, emphasizing the importance of addressing availability to enhance provision

Despite this association, the weak Cramer’s V value suggests that availability alone is not sufficient to guarantee provision. This discrepancy was evident in services such as post-abortion care (PAC) and support for survivors of physical and sexual abuse, which were often inadequately provided despite being reported as available. For PAC, many primary healthcare facilities in Nigeria lack the capacity to offer comprehensive care. Bell et al. [34] found that less than half of facilities in Nigeria provide basic PAC services, and only 23.9% offer comprehensive care. Hesitation based on moral/religious grounds among providers further compound this issue, even though PAC is legal in Nigeria [35]

Similarly, care for survivors of sexual and physical abuse remains insufficient, with significant barriers in accessing these services. Most primary healthcare providers lack the training to respond effectively to sexual and gender-based violence [36]. Survivors often face stigmatizing attitudes and victim-blaming from healthcare workers, discouraging them from seeking care [37]. O’Dwyer, Tarzia, and Fernbacher [38] highlighted that many healthcare workers are unprepared to handle trauma-sensitive cases, leading to unsympathetic or dismissive responses that further alienate survivors

## Conclusion

The availability and provision of quality SRH services are essential for improving health outcomes, especially in rural areas where access disparities are most pronounced. While education and training of healthcare providers enhance both the range and effectiveness of services offered, sensitive services such as post-abortion care and support for abuse survivors remain grossly inadequate. Based on these findings, it is crucial to enhance the competency of healthcare providers in delivering complex SRH services by investing in their education and training. Strengthening the role of community health workers (CHWs) by providing them with adequate resources and support will ensure effective service provision extending to other cadres of health workers. Implementing holistic training approaches that include continuous mentorship, supportive supervision, and community engagement will help address cultural and religious barriers. Additionally, improving infrastructure and resource allocation is essential to ensure the comprehensive availability and provision of SRH services, particularly post-abortion care (PAC) and support for survivors of physical and sexual abuse.

This study’s cross-sectional design limits causal interpretation, meaning observed associations between provider characteristics and service outcomes cannot confirm cause-and-effect relationships. The reliance on self-reported data may introduce social desirability or recall bias, especially for sensitive topics like youth sexual and reproductive health services. Although several covariates were adjusted for in the analysis, unmeasured factors such as provider attitudes, community norms, or facility-level resources may still confound the findings. Importantly, while clustering was accounted for during the analysis phase, it was not considered during sample size calculation, which may affect the statistical power and precision of the estimates. While these limitations should be acknowledged, they do not diminish the value of the findings. Instead, they highlight areas for further research and provide a foundation for more nuanced policy and programmatic decisions moving forward.

## Supplementary Information

Supplementary Material 1.

## Data Availability

The data supporting the findings of this study are available from the Health Policy Research Group (HPRG), University of Nigeria. However, due to access restrictions, these data are not publicly available. They can be made available upon reasonable request from the corresponding author, Joy Nkeiruka Ozughalu, with prior approval from HPRG.

## Abbreviations

SRH: Sexual and Reproductive Health
PHCs: Primary Healthcare Centers
LGAs: Local Government Areas
AYP: Adolescent and Young People
STIs: Sexually Transmitted Infections
HIV: Human Immunodeficiency Virus
MNCH: Maternal, Newborn, and Child Health
PHCUOR: Primary Health Care Under One Roof
PAC: Post Abortion Care
HSAF: Health Service Availability in Facility
HSPF: Health Service Priovision in Facility

## References

[1] United Nations Population Fund. World Population Dashboard. Nigeria. https://www.unfpa.org/data/world-population-dashboard. Accessed 21 Jun 2023.

[2] Obiezu -Umeh C, Nwaozuru U, Mason S, et al. Implementation strategies to enhance youthfriendly sexual and reproductive health services in subsaharan africa: a systematic review. Front Reprod Health. 2021;3:684081. 10.3389/frph.2021.684081 36304027 10.3389/frph.2021.684081 PMC9580831

[3] Brunelli L, Bravo G, Romanese F, et al. Sexual and reproductive healthrelated knowledge, attitudes, and support network of Italian adolescents. Public Health Pract. 2022;3:100253. 10.1016/j.puhip.2022.100253. 10.1016/j.puhip.2022.100253 PMC9461229 36101775

[4] Mihretie G, Goshu Y, Belay H, et al. Sexual and reproductive health issues and associated factors among female night school students in Amhara region, ethiopia: an institutionbased crosssectional study. BMJ Open. 2023;13(7):e066244. 10.1136/bmjopen-2022-066244. 37407060 10.1136/bmjopen-2022-066244 PMC10335416

[5] Adewole I, Primary Health Care. Thirty-six States and the FCT are to Share $1.5m FG Fund for. 2016. https://www.informationng.com/2016/07/36-states-and-the-fct-to-share-1-5m-fg-fund-for-primary-healthcare.htm. Accessed 28 May 2025.

[6] National Primary Health Care Development Agency. Annual report on primary health care functionality in Nigeria. Abuja, Nigeria: NPHCDA; 2020.

[7] Agu I, Agu C, Mbachu C, Onwujekwe O. Impact of a capacitybuilding intervention on views and perceptions of healthcare providers towards the provision of adolescent sexual and reproductive health services in Southeast nigeria: a crosssectional qualitative study. BMJ Open. 2023;13. 10.1136/bmjopen-2023-073586. 10.1136/bmjopen-2023-073586 PMC10679974 38000827

[8] Adione AA, Abamara N, Vivalya BMN. Determinants of the utilization of youthfriendly sexual and reproductive health services in public secondary schools of Kogi state, nigeria: an explorative study. BMC Public Health. 2023;23:1091. 10.1186/s12889-023-15926-y. 37280546 10.1186/s12889-023-15926-y PMC10243027

[9] Nmadu AG, Mohammed S, Usman NO. Barriers to adolescents’ access and utilisation of reproductive health services in a community in Northwestern nigeria: a qualitative exploratory study in primary care. Afr J Prim Health Care Fam Med. 2020;12(1):a2307. 10.4102/phcfm.v12i1.2307. 10.4102/phcfm.v12i1.2307 PMC7433241 32787401

[10] Isaruk ID, Isaruk JID, George DT. Attitude and ethical behaviors of healthcare providers as antidotes of health service consumer satisfaction in Mgbuoshimini primary health centre, Port harcourt, Nigeria. J Health Appl Sci Manag. 2023. 10.4314/johasam.v6i3.4.

[11] Mbachu CO, Agu IC, Eze I, et al. Exploring issues in caregivers and parent communication of sexual and reproductive health matters with adolescents in Ebonyi state, Nigeria. BMC Public Health. 2020;20(1):77. 10.1186/s12889-019-8058-5. 31952497 10.1186/s12889-019-8058-5 PMC6969441

[12] Envuladu EA, Massar K, de Wit J. Healthcare workers’ delivery of adolescent responsive sexual and reproductive healthcare services: an assessment in plateau state, Nigeria. BMC Womens Health. 2023;23:132. 10.1186/s12905-023-02288-1. 36966291 10.1186/s12905-023-02288-1 PMC10040103

[13] United Nations Population Fund. State of the world population 2022. New York, NY: UNFPA; 2022. 10.18356/9789210021537.

[14] Ebonyi State Government. Population figure. 2023. https://www.ebonyistate.gov.ng/profile.html. Accessed 21 Jun 2023.

[15] EbonyiNational Youth Baseline Survey. National Youth Baseline Survey Nigeria Data Portal. Published 20 Mar 2014. https://opendataforafrica.org. Accessed 21 Jun 2023.

[16] Odo AN, Samuel ES, Nwagu EN, Nnamani PO, Atama CS. Sexual and reproductive health services (SRHS) for adolescents in Enugu state, Nigeria: a mixed methods approach. BMC Health Serv Res. 2018;18(1):1. 10.1186/s12913-017-2779-x. 29422062 10.1186/s12913-017-2779-x PMC5806240

[17] Singh KA, Verma R, Barker G. Making women count: an annual publication on gender and evaluation by UN women multi country office for India, Bhutan, Sri Lanka and Maldives. New Delhi, India: UN Women; 2013.

[18] Nanda G. Compendium of gender scales., Washington DC. FHI 360/CChange; 2011. https://sbccimplementationkits.org/demandrmnch/ikitresources/compendium-of-gender-scales-3/. Accessed 21 Jun 2023.

[19] Millogo O, Doamba JEO, Sié A, Utzinger J, Vounatsou P. Constructing a malariarelated health service readiness index and assessing its association with child malaria mortality: an analysis of the Burkina Faso 2014 SARA data. BMC Public Health. 2021;21(1):20. 10.1186/s12889-020-09994-7. 33402160 10.1186/s12889-020-09994-7 PMC7784320

[20] Ugwu G, Enebe N, Onah C, et al. Primary health care under one roof: knowledge and predictors among primary health care workers in Enugu state, South east, Nigeria. Niger J Med. 2020;29(4):649–54.

[21] Findley S, Uwemedimo O, Doctor H, et al. Early results of an integrated maternal, newborn, and child health program, Northern nigeria, 2009 to 2011. BMC Public Health. 2013;13:1034. 10.1186/1471-2458-13-1034. 24175944 10.1186/1471-2458-13-1034 PMC4228462

[22] Okonofua FE, Ntoimo LF, Yaya S, Igboin B, Solanke O, Ekwo C, et al. Effect of a multifaceted intervention on the utilisation of primary health for maternal and child health care in rural nigeria: a quasiexperimental study. BMJ Open. 2022;12. 10.1136/bmjopen-2021-049499. 10.1136/bmjopen-2021-049499 PMC8830217 35135763

[23] Callaghan TH, Falia G, Greer E, Ramy M, Washburn DJ. Analysis of an innovative approach to target rural communities in public health funding. 2023. 10.21423/1969.1/201257

[24] Nyblade L, Stockton MA, Giger K, et al. Stigma in health facilities: why it matters and how we can change it. BMC Med. 2017;15(1):25. 10.1186/s12916-017-0783-8. 30764806 10.1186/s12916-019-1256-2 PMC6376713

[25] Jonas K, Crutzen R, Krumeich AJ, et al. Healthcare workers’ behaviors and the role of education in promoting health service delivery. Int J Health Policy Manag. 2017;6(9):515–22. 10.15171/ijhpm.2017.22.

[26] Babalola S, Fatusi A, Anyanti J. The effect of education on health outcomes in rural nigeria: a focus on SRH services. Health Policy Plan. 2016;31(7):875–84. 10.1093/heapol/czw013.

[27] Ayuk BE, Yankam BM, Saah FI, Bain LE. Provision of injectable contraceptives by community health workers in subsaharan africa: a systematic review of safety, acceptability, and effectiveness. Hum Resour Health. 2022;20. 10.1186/s12960-022-00763-8. 10.1186/s12960-022-00763-8 PMC9446834 36064408

[28] Jisso M, Feyasa MB, Medhin G, et al. Sexual and reproductive health service utilization of young girls in rural ethiopia: what are the roles of health extension workers? Communitybased crosssectional study. BMJ Open. 2022;12. 10.1136/bmjopen-2021-056639. 10.1136/bmjopen-2021-056639 PMC9494555 36130743

[29] Kyomuhangi T, Naggayi B, Turyakira E, et al. Understanding complex community structures to improve sexual reproductive health for adolescents in Uganda. Paediatr Child Health. 2021;26:e60. 10.1093/pch/pxab061.065.

[30] Campbell OM, Calvert C, Testa A, et al. The role of education in mitigating SRH barriers for health workers in subsaharan Africa. Reprod Health. 2016;13(1):74. 10.1186/s12978-016-0198-7.27305901

[31] Abiodun OM, Balogun OR, Adepeju MM. The impact of health worker education on SRH service delivery in Nigerian healthcare facilities. Afr Health Sci. 2019;19(1):1468–75. 10.4314/ahs.v19i1.40.

[32] Bintabara D, Ernest A, Mpondo B. Health facility service availability and readiness to provide basic emergency obstetric and newborn care in a lowresource setting: evidence from a Tanzania National survey. BMJ Open. 2019;9(2):e020608. 30782861 10.1136/bmjopen-2017-020608 PMC6398731

[33] Tumwine G, Palmieri J, Larsson M, et al. Understanding healthcare practitioners’ perceptions and behaviors towards SRH rights in lowresource settings. PLoS ONE. 2020;15(6):e0234658. 32584840 10.1371/journal.pone.0234658 PMC7316327

[34] Bell S, Shankar M, Ahmed S, et al. Postabortion care availability, facility readiness and accessibility in Nigeria and Côte d’ivoire. Health Policy Plan. 2021;36:1077–89. 10.1093/heapol/czab068. 34131700 10.1093/heapol/czab068 PMC8359750

[35] Onah H, Ogbuokiri C, Obi S, et al. Knowledge, attitude, and practice of private medical practitioners towards abortion and postabortion care in enugu, southeastern Nigeria. J Obstet Gynaecol. 2009;29(5):415–8. 10.1080/01443610902918613. 19603321 10.1080/01443610902918613

[36] Ikuteyijo O, KaiserGrolimund A, Fetters M, et al. Health providers’ response to female adolescent survivors of sexual and genderbased violence and demandside barriers in the utilization of support services in urban lowincome communities of Nigeria. Healthcare. 2023;11(19). 10.3390/healthcare11192627. 10.3390/healthcare11192627 PMC10572492 37830664

[37] Pijlman V, Eichelsheim V. Sometimes it seems easier to push it away: a study into the barriers to helpseeking for victims of sexual violence. J Interpers Violence. 2023;38(5):316–33. 10.1177/08862605221147064. 10.1177/08862605221147064 36710513

[38] O’Dwyer C, Tarzia L, Fernbacher S. Health professionals’ experiences of providing care for women survivors of sexual violence in psychiatric inpatient units. BMC Health Serv Res. 2019;19(1):1–11. 10.1186/s12913-019-4683-z. 31727056 10.1186/s12913-019-4683-z PMC6857150

