# Supplementary material for: Exploring the patterns of availability and provision of sexual and reproductive health services to young people in primary healthcare centers in Ebonyi state, Nigeria
Source: BMC Health Serv Res. 2025 Aug 11;25:1061. doi: 10.1186/s12913-025-13208-4 (PMC12341132; doi:10.1186/s12913-025-13208-4)
Supplement: Supplementary file 1 — Supplementary Material 1. [file 12913_2025_13208_MOESM1_ESM.docx]

**Improving youth-friendly SRHR services through gender-transformative and intersectional approaches using community-embedded interventions in Ebonyi State**

**Health Service Provider Questionnaire**

## **Introduction, purpose, and procedure**

Good morning/afternoon, Sir/Ma. My name is ___________________ and I am a trained data collector from Health Policy Research Group University of Nigeria Enugu Campus.

We are working with the Ebonyi State government to implement a research project that aims to make sexual and reproductive health services more gender equitable and inclusive for young people (aged 10-24 years) in Ebonyi State. To achieve this aim, we wish to conduct a survey to understand the gender norms and other societal factors that influence the provision of sexual and reproductive health services for young people in this community.

You will get the results of this exercise in the future and we would use your responses to help you and the government to improve provision and use of SRHS for young people. Please answer to the best of your capability since your responses will influence how the government will respond to the improvement of SRHS in this area. Your participation is completely voluntary, and you do not have to answer any questions you do not want to answer.

Health service providers were randomly selected from the six LGAs where this programme is being implemented and you happen to be one of them.

I would now like to ask you a few questions that should not take more than 45 minutes. I will not write down your name and everything you tell me will be kept strictly confidential. You do not have to participate in this interview, and if you choose to be interviewed, you do not have to answer every question I ask you.

Do you have any questions about this?

## **Background information**

| **LGA CODE** | \|___\|___\| |
| --- | --- |
| **HEALTH FACILITY NAME** | ________ |
| **RURAL OR URBAN CODE** | [____]____\| |
| **INTERVIEWER CODE** | [____]____\| |
| **RESPONDENT’S NUMBER** | [____]____] |
| **RESPONDENT’S CODE** | LGA + COMMUNITY + RUR/URB + INTERVIEWER + RESPONDENT  [___[__­­­_] + [____[___] + [___]___] + [____]____] + [____]____] |
| **DATE AND START TIME**  **OF INTERVIEW** | DD MM YYYY HR MIN  [___\|___] / [___\|___] / [___\|___]___]___] / [____\|____] |
| **GPS LOCATION** |  |

## **Section 1: Demographic and socio-economic characteristics of respondents**

| **S/N** | **Questions** | **Coding categories** | **Response** | **Skip/Filter** |
| --- | --- | --- | --- | --- |
|  | Sex of respondent  *(Observe and record, but do not ask)* | Female = 1  Male = 0 | [___] |  |
|  | How old are you? *(Age at your last birthday)* |  | [___] |  |
|  | What is your current role as a healthcare provider? | Officer-in-charge = 1  Adolescent health focal officer = 2  Frontline health worker in youth-friendly centers = 3  Community health worker = 4  Youth counsellor = 5 | [___] |  |
|  | How long have you had this role? |  | [___] |  |
|  | Where are you located? | Town centre =1  Market =2  Village centre =3  Outskirts of town/village =4  Other =5 | [___]  [___]  [___]  [___]  [___] |  |
|  | Were you trained for the type of work you do? | Yes = 1  No = 2 | [___] |  |
|  | How many years of formal education did you receive for the work you do? | ___________ | [___] |  |
|  | In total, how many years of formal education did you receive? | ___________ | [___] |  |
|  | Have you received any formal training on the provision of youth-friendly sexual and reproductive health services? | Yes = 1  No = 2 | [___] |  |

## **Section 2: Experiences of providing youth-friendly sexual and reproductive health services**

|  | | | | |
| --- | --- | --- | --- | --- |
|  | Which of the following health services are available in this facility for young people? | Yes = 1; No = 2   1. Information and counseling on reproductive health, sexuality & safe sex 2. HIV testing and counseling 3. HIV care and support 4. Diagnosis of STI/RTI 5. Treatment and counseling for STIs/RTIs 6. Pregnancy testing 7. Care during pregnancy 8. Care during childbirth 9. Care after childbirth 10. Post-abortion care 11. Information & counseling on contraception/emergency contraception 12. Information and counseling on condoms and condom use 13. Supply of contraceptives/condoms 14. Care and support for physical abuse 15. Care and support for sexual abuse (rape)   Others (specify) ……………… 99 | [___]  [___]  [___]  [___]  [___]  [___]  [___]  [___]  [___]  [___]  [___]  [___]  [___]  [___]  [___] |  |
|  | Which of the following health services have you provided for young people in this PHC or elsewhere? | Yes = 1; No = 2   1. Information and counselling on reproductive health, sexuality & safe sex 2. HIV testing and counseling 3. HIV care and support 4. Diagnosis of STI/RTI 5. Treatment and counseling for STIs/RTIs 6. Pregnancy testing 7. Care during pregnancy 8. Care during childbirth 9. Care after childbirth 10. Post-abortion care 11. Information & counseling on contraception/emergency contraception 12. Information and counseling on condoms and condom use 13. Supply of contraceptives/condoms 14. Care and support for physical abuse 15. Care and support for sexual abuse (rape)   Others (specify) ……………… 99 | [___]  [___]  [___]  [___]  [___]  [___]  [___]  [___]  [___]  [___]  [___]  [___]  [___]  [___]  [___] |  |
|  | Do you treat girls/young women differently from boys/young men when they seek for sexual or reproductive health services? | Yes = 1  No = 2 | [___] |  |
|  | Do you treat young people differently from adults when they seek for sexual or reproductive health services? | Yes = 1  No = 2 | [___] |  |
